# Maximum Correntropy Unscented Kalman Filter for Ballistic Missile Navigation System based on SINS/CNS Deeply Integrated Mode

**DOI:** 10.3390/s18061724

**Published:** 2018-05-27

**Authors:** Bowen Hou, Zhangming He, Dong Li, Haiyin Zhou, Jiongqi Wang

**Affiliations:** 1 Department of System Science, College of Liberal Arts and Science, National University of Defense Technology, Fuyuan Road No.1, Changsha 410072, China; houbowen12@nudt.edu.cn (B.H.); hzmnudt@sina.com (Z.H.); gfkd_zhy@sina.com (H.Z.); 2 Beijing Institute of Control Engineering, China Academy of Space Technology, Beijing 100080, China; 3 Unit 94, PLA 91550, Dalian 116023, China; nudtlidong@163.com

**Keywords:** unscented Kalman filter, maximum correntropy, SINS/CNS deeply integrated navigation, non-Gaussian noises, ballistic missile

## Abstract

Strap-down inertial navigation system/celestial navigation system (SINS/CNS) integrated navigation is a high precision navigation technique for ballistic missiles. The traditional navigation method has a divergence in the position error. A deeply integrated mode for SINS/CNS navigation system is proposed to improve the navigation accuracy of ballistic missile. The deeply integrated navigation principle is described and the observability of the navigation system is analyzed. The nonlinearity, as well as the large outliers and the Gaussian mixture noises, often exists during the actual navigation process, leading to the divergence phenomenon of the navigation filter. The new nonlinear Kalman filter on the basis of the maximum correntropy theory and unscented transformation, named the maximum correntropy unscented Kalman filter, is deduced, and the computational complexity is analyzed. The unscented transformation is used for restricting the nonlinearity of the system equation, and the maximum correntropy theory is used to deal with the non-Gaussian noises. Finally, numerical simulation illustrates the superiority of the proposed filter compared with the traditional unscented Kalman filter. The comparison results show that the large outliers and the influence of non-Gaussian noises for SINS/CNS deeply integrated navigation is significantly reduced through the proposed filter.

## 1. Introduction

With the development of missile technology, even higher accuracy of navigation is requested. Single navigation mode cannot satisfy the application requirement. Because of the uncertainty in the actual navigation system, a number of algorithms have been designed to improve the accuracy of state estimation [1]. To sum up, the navigation accuracy of ballistic missile has focused on two parts, navigation system construction and navigation filter algorithm designation.

For navigation system construction, a number of navigation modes have been designed to improve navigation accuracy. Among these modes, the strap-down inertial navigation system (SINS) has been playing an important role among navigation systems for several decades [2,3,4]. It can autonomously achieve a navigation mission completely relying on micro electro mechanical systems (MEMS) and has a strong anti-interference capability. Moreover, the attitude, the velocity, and the position of ballistic missiles can be easily obtained by its output information. However, the accumulated navigation errors caused by the inertial components cannot be avoided, especially in the long-term missile navigation [5]. Therefore, in the practical situations, some other supporting facilities, including the Global Navigation Satellite System (GNSS), BeiDou Navigation Satellite System (BDS), the Doppler Velocity Log (DOL), and the celestial navigation system (CNS), are always integrated with SINS for the purpose of improving navigation accuracy [6,7]. However, GNSS is always susceptible to interference, which will have a strong impact on missile performance. DOL is always used underwater and is limited by the distance, which is not appropriate for long-distance missiles. The CNS is a navigation system that calculates the accurate attitude on the basis of measuring the azimuth of a celestial body through the celestial sensor. Because the source of information in the CNS is a celestial body, it has many merits, such as good concealment, strong autonomy, and high navigation accuracy, but the output frequency is much lower and discontinuous, and the output information can be easily influenced by surroundings [8,9,10]. Hence, the CNS is frequently used for the purpose of assisting the SINS in the aerospace field, utilizing the SINS/CNS integrated navigation system. The SINS/CNS integrated navigation system can correct the attitude error and the gyro drift using the starlight information and greatly improve navigation accuracy [11]. At present, it is an important developing direction for missile, airplane, and spacecraft navigation technology [12]. Nevertheless, the traditional system only makes use of the information of star sensors to reckon the gyro drift, but it cannot estimate the accelerometer bias, which leads to position divergence. Therefore, some improved SINS/CNS integrated navigation systems have been proposed [4,13,14]. They make use of more astronomical observation information to realize SINS/CNS deeply integrated navigation systems and effectively improve their performance.

For navigation filter design, many algorithms have been applied to the navigation system. The Kalman filter (KF) is the most popular optimal state estimation method used for the linear dynamic system [15]. However, for navigation systems, the navigation system equation is always nonlinear [16]. The frequently used nonlinear algorithms for solving nonlinear problems mainly include the extended Kalman filter (EKF) [17] and the unscented Kalman filter (UKF) [18]. EKF is a popular nonlinear extension of the KF, which uses Taylor series expansions to approximate the nonlinear system. However, the Taylor series expansions in many navigation systems are cumbersome and always lead to implementation difficulties [15]. The iterated extened Kalman filter (IEKF) overcomes the problem and improves the accuracy with the Gauss-Newton method [19]. Furthermore, the nonlinear iterated filter (NIF) improves the stability of the Jacobian matrix in the EKF and the IEKF through the Levenberg–Marquardt algorithm [20]. In the UKF, a set of selected sigma points are used to approximate the probability distribution function of the state and propagate through the nonlinear process and the measurement equation. Thus, the UKF saves the trouble of calculating the Taylor series is more accurate than the EKF. The posterior linearization filter (PLF), using statistical linear regression, is a newly proposed algorithm to improve the filter accuracy based on sigma-point approximations [21]. However, the filters above are always used to deal with Gaussian white noise. Nevertheless, owing to the operational error or the complex atmospheric environment, the non-Gaussian noises, such as large outliers and Gaussian mixture noises, often exist during the actual navigation process.

For non-Gaussian process, many nonlinear filters do not perform satisfactorily [22]. However, the filter robustness can be improved through the approaches as follows:

1. A classical method involves creating filters directly for the systems under the conditions of non-Gaussian noises to deal with noises with different heavy-tailed distributions [23,24]. However, it is difficult to take effect in multidimensional situations, which limits its applicability [25].
2. There are two efficient methods to handle non-Gaussian noises based on estimating the a posteriori probability density with a series of samples. The UKF approximates the mean and the covariance of the state estimation using unscented transformation (UT) through sigma points [26]. The ensemble Kalman filter (EnKF) is another algorithm to capture state estimation using samples set for the purpose of handling non-Gaussian noises [27].
3. Another popular method is the Huber-based Kalman filter. It is a robust state estimator to handle the non-Gaussian noises based on the robust estimation theory [28]. Many navigation and target problems were often handled through it under the conditions of non-Gaussian noises in the past few years [29,30,31,32].
4. Chang [33] proposed a new robust Kalman filter in recent years. He defined a judging index calculating the difference between the observation and the prediction based on Mahalanobis distance to solve the outliers, which can also successfully solve the observation noises that obey the thick-tailed distribution during the actual observation process.

Maximum correntropy criterion (MCC) is an information theoretic method proposed in recent years. It has been applied to the Bayesian estimation and the error analysis [34,35]. The maximum correntropy Kalman filter (MCKF), which is an improved Kalman filter algorithm using the MCC theory, is one of the latest designed estimators for the purpose of solving non-Gaussian noises. In addition, the maximum correntropy extended Kalman filter (MCEKF) and the maximum correntropy unscented Kalman filter (MCUKF) are proposed for the nonlinear system [36], and the MCUKF has been proven to show a better performance than MCEKF [37]. However, its robustness is not analyzed and it has not been applied to SINS/CNS deeply integrated navigation.

The key contributions of this paper are expressed as follows. First of all, the observability of the new deeply integrated navigation system is analyzed. The MCUKF is then deduced on the basis of maximum correntropy theory and unscented transformation to deal with the nonlinearity and non-Gaussian noises in the navigation system. The computational complexity of it is also analyzed. Furthermore, the robustness of the MCUKF is discussed under the condition of the non-Gaussian noises. Finally, the conditions of non-Gaussian noise, mainly including the large outliers and the Gaussian mixture noises, existing in SINS/CNS deeply integrated navigation are effectively handled by the MCUKF.

The organization of this paper proceeds as follows. SINS/CNS deeply integrated navigation is briefly reviewed and the observability is analyzed in Section 1. In Section 2, the algorithm steps are designed for the MCUKF and the robustness performance is analyzed compared with a Huber-based filter. In Section 3, simulations are demonstrated to compare the performances using several filter methods under the conditions of different kinds of noises. Finally, we draw conclusions in Section 4.

## 2. SINS/CNS Deeply Integrated Navigation System

### 2.1. Integrated Navigation Principle

**Figure 1:**
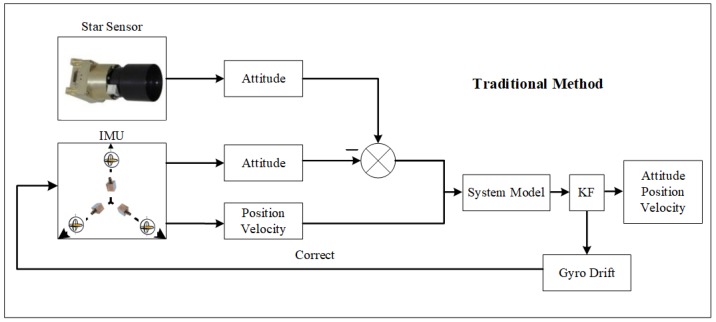
Traditional strap-down inertial navigation system/celestial navigation system (SINS/CNS) integrated navigation principle.

The traditional SINS/CNS integrated navigation system outputs the attitude, the velocity, and the position information of a missile through the IMU (including a gyro and an accelerometer). The star sensor of the CNS outputs the attitude information of the missile after star capturing and star identification. The attitude information calculated by the star sensor and the gyro is then integrated, and the integrated information is served as the measurement equation. The optimal estimation methods are then used to estimate the gyro drift to correct the gyro. Figure 1 shows the traditional SINS/CNS integrated navigation principle.

**Figure 2:**
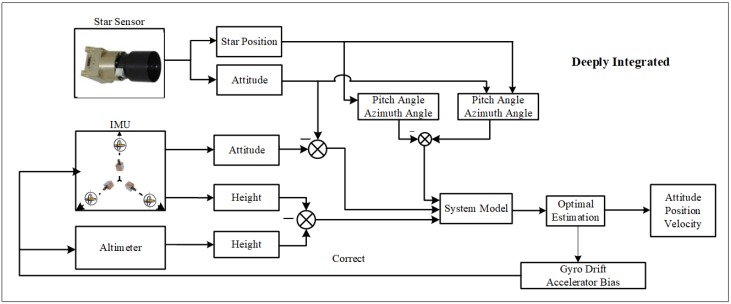
SINS/CNS deeply integrated navigation principle.

Although SINS/CNS integrated navigation system can correct the gyro drift to some extent, the error of the state variables still has a divergence [4,13,14,16]. SINS/CNS deeply integrated navigation can solve the problem. Based on the traditional integrated navigation system, the height, the pitching angle, and the azimuth angle are added as the measurement information according to the altimeter and the astronomical information. In fact, these three new measurements are the function of the position of the navigation target. Therefore, the estimation of the gyro drift and the accelerometer bias can be gained to correct the acceleration information of SINS. Figure 2 shows the navigation principle of the SINS/CNS deeply integrated mode.

The deeply integrated SINS/CNS integrated navigation system has not yet been realized in real scenarios. When it is applied on the missile, the star sensor measures not only the attitude information but also the partial position information of the missile. The attitude can be calculated through star identification. The position can be calculated by measuring the height angle and the azimuth angle of the star. An altimeter needs to be installed on the system to measure the height information of the missile. The altimeter can be a radar altimeter [38], a pressure altimeter [39], or a laser altimeter [40], decided according to the specific case. Combined with the position information from the star sensor, the position of the missile can be obtained. In this way, the deeply SINS/CNS integrated navigation system can be realized and applied to practical situations. The detailed principle of the system is introduced in the next section.

### 2.2. System Model

#### 2.2.1. State Equation

Suppose that the navigation frame is the launch point frame (l-frame) and the SINS is strapdown-installed along the three axles of the missile. Then the state equation is identical to the traditional algorithm:

(1) $$\dot{X}\left(t\right)=F\left(t\right)X\left(t\right)\;+\;G\left(t\right)W\left(t\right),$$

where $X\left(t\right)={\left[\begin{array}{ccccc} \Psi (t) & \delta v(t) & \delta r_{l}\left(t\right) & \varepsilon (t) & \nabla \end{array}\left(t\right)\right]}^{T}$ denotes the system state, $\Psi \left(t\right)=\left[\begin{array}{ccc} \phi_{x}\left(t\right) & \phi_{y}\left(t\right) & \phi_{z}\left(t\right) \end{array}\right]$ denotes the platform angles error (the navigation frame misalignment angle) in the l-frame; $\delta v\left(t\right)=\left[\begin{array}{ccc} \delta v_{xl}\left(t\right) & \delta v_{yl}\left(t\right) & \delta v_{zl}\left(t\right) \end{array}\right]$ denotes the velocity error in the l-frame; $\delta r_{l}\left(t\right)=\left[\begin{array}{ccc} \delta x_{l}\left(t\right) & \delta y_{l}\left(t\right) & \delta z_{l}\left(t\right) \end{array}\right]$ denotes the position error in the l-frame; $\varepsilon \left(t\right)=\left[\begin{array}{ccc} \varepsilon_{x}\left(t\right) & \varepsilon_{y}\left(t\right) & \varepsilon_{z}\left(t\right) \end{array}\right]$ denotes the gyro constant drift; $\nabla \left(t\right)=\left[\begin{array}{ccc} \nabla_{x}\left(t\right) & \nabla_{y}\left(t\right) & \nabla_{z}\left(t\right) \end{array}\right]$ denotes the accelerometer constant bias; $W\left(t\right)={\left[\begin{array}{cccccc} \varepsilon_{sx}\left(t\right) & \varepsilon_{sy}\left(t\right) & \varepsilon_{sz}\left(t\right) & \nabla_{sx}\left(t\right) & \nabla_{sy}\left(t\right) & \nabla_{sz}\left(t\right) \end{array}\right]}^{T}$ denotes the process noise vector, including the random noises of the gyro and the accelerometer; G(t) denotes the process noise input matrix; F(t) denotes the process input matrix. The detailed information of F(t) and G(t) can be found in [41]— Equations (6-5) and (6-6), respectively. The solution of the differential Equation (1) for the discrete mode can be expressed as:

(2) $$X\left(k\right)=f\left(X\left(k-1\right),W\left(k-1\right)\right),$$

Equation (2) can be obtained through the Laplace transformation which is introduced in Chapter 2 of Reference [41]. In simulation, the equation is solved through Runge-Kutta algorithm.

And the linear version for Equation (2), which is used for MCEKF [36] can be expressed as:

(3) $$X\left(k\right)=\Phi \left(k-1\right)X\left(k-1\right)\;+\;\Gamma \left(k-1\right)W\left(k-1\right),$$

where *k* denotes the epoch, $\Phi \left(k-1\right)$ and $\Gamma \left(k-1\right)$ are the discrete versions of $f\left(X\left(k-1\right)\right)$ and $G\left(k-1\right)$, respectively, which can be expressed as:

(4) $$\begin{array}{c} \Phi \left(k-1\right)=I+F\left(k-1\right)T+\frac{1}{2!}F^{2}\left(k-1\right)T^{2}\\ \Gamma \left(k-1\right)=T\left(I+\frac{1}{2!}F\left(k-1\right)T+\frac{1}{3!}F^{2}\left(k-1\right)T^{2}\right)G\left(k\right). \end{array}$$

#### 2.2.2. Measurement Equation

**(1) Measurement of Attitude Error**

Suppose that the attitude output of the gyro is $\theta_{g}\left(k\right)={\left[\begin{array}{ccc} \theta_{g}\left(k\right) & \varphi_{g}\left(k\right) & \psi_{g}\left(k\right) \end{array}\right]}^{T}$, and the attitude output of the star sensor is $\theta_{s}\left(k\right)={\left[\begin{array}{ccc} \theta_{s}\left(k\right) & \varphi_{s}\left(k\right) & \psi_{s}\left(k\right) \end{array}\right]}^{T}$. The three angles are equivalent to the three Euler angles, denoting roll angle, pitch angle, and yaw angle, respectively. The difference between the two sets of attitude angles can be denoted as $\delta \theta \left(k\right)=\theta_{g}\left(k\right)-\theta_{s}\left(k\right)$, which can be transformed into the error-angles of the platform and can be expressed as the function of the state Ψ(k). Denote $Z_{1}\left(k\right)=\delta \theta \left(k\right)$ as the attitude error measurement, which can be expressed as:

(5) $$Z_{1}\left(k\right)=M\left(k\right)X\left(k\right)+V_{1}\left(k\right),$$

where $M\left(k\right)=\left[\begin{array}{cc} M_{0}\left(k\right) & O_{3\;\times \;12} \end{array}\right]$, and $M_{0}\left(k\right)$ denotes the attitude error transition matrix, which can be expressed as:

(6) $$M_{0}\left(k\right)=\left[\begin{array}{ccc} 0 & \cos\varphi (k) & -\cos\psi (k)\sin\varphi (k)\\ 0 & \sin\varphi (k) & \cos\psi (k)\cos\varphi (k)\\ 1 & 0 & \sin\psi (k) \end{array}\right],$$

where φ(k) denotes the pitch angle, ψ(k) denotes the yaw angle, and $V_{1}\left(k\right)$ represents the measurement noises of the star sensor. The detailed derivation process of $M_{0}\left(k\right)$ can be obtained in [42].

**(2) Measurement of Position Error**

The measurement information relies on the azimuth angle, the pitch angle of the guide star, and the outputs of the altimeter. The measurement equation of the position error is derived in [4]. The measurement equation is expressed as:

(7) $$\left[\begin{array}{c} \delta P(k)\\ \delta A(k)\\ \delta h(k) \end{array}\right]=M_{1}\left(k\right){\left(C_{i}^{l}C_{e}^{i}\left(k\right)M_{2}\left(k\right)\right)}^{-1}\left[\begin{array}{c} \delta x_{l}\left(k\right)\\ \delta y_{l}\left(k\right)\\ \delta z_{l}\left(k\right) \end{array}\right]+\left[\begin{array}{c} v_{P}\left(k\right)\\ v_{A}\left(k\right)\\ v_{h}\left(k\right) \end{array}\right],$$

where δP(k) is the pitch angle difference between the measurement and the computation value; δA(k) is the azimuth angle difference between the measurement and the computation value; δh(k) is the difference between the height measurement of the altimeter and the computation value; $C_{i}^{1}$ stands for the rotation matrix from the l-frame (the launching point coordinate frame which is also the navigation frame) to the i-frame (the geocentric inertial coordinate frame), which is a constant matrix and can be expressed as:

(8) $$C_{i}^{l}=\left[\begin{array}{ccc} -\cos\zeta \sin\gamma_{0}\cos\phi_{0}-\sin\zeta \sin\phi_{0} & \cos\gamma_{0}\cos\phi_{0} & \sin\zeta \sin\gamma_{0}\cos\phi_{0}-\cos\zeta \sin\phi_{0}\\ -\cos\zeta \sin\gamma_{0}\sin\phi_{0}+\sin\zeta \cos\phi_{0} & \cos\gamma_{0}\sin\phi_{0} & \sin\zeta \sin\gamma_{0}\sin\phi_{0}+\cos\zeta \cos\phi_{0}\\ \cos\zeta \cos\gamma_{0} & \sin\gamma_{0} & -\sin\zeta \cos\gamma_{0} \end{array}\right],$$

where ζ denotes the launching angle of missile, $\phi_{0}$ and $\gamma_{0}$ denote the longitude and the latitude of the launching point, respectively, $C_{e}^{i}\left(k\right)$ denotes the rotation matrix from the i-frame to the e-frame (the earth-based frame), which can be expressed as:

(9) $$C_{e}^{i}\left(k\right)=\left[\begin{array}{ccc} \cos\omega_{e}t(k) & \sin\omega_{e}t(k) & 0\\ -\sin\omega_{e}t(k) & \cos\omega_{e}t(k) & 0\\ 0 & 0 & 1 \end{array}\right],$$

where $\omega_{e}$ is the earth’s rotation angular velocity, t(k) denotes the time in the k-th epoch. $\delta r_{l}\left(t\right)=\left[\begin{array}{ccc} \delta x_{l}\left(k\right) & \delta y_{l}\left(k\right) & \delta z_{l}\left(k\right) \end{array}\right]$ denotes the position error in three directions; $v_{P}\left(k\right)$ and $v_{A}\left(k\right)$ are the observation noises of the pitch angle and the azimuth angle, respectively; $v_{h}\left(k\right)$ is the measurement noise of the altimeter; $M_{1}\left(k\right)$ and $M_{2}\left(k\right)$ are as follows:

(10) $$M_{1}\left(k\right)=\left[\begin{array}{ccc} -\cos A(k) & -\sin A\left(k\right)\cos\varphi_{b}\left(k\right) & 0\\ -\tan P(k)\sin A(k) & \tan P\left(k\right)\cos A\left(k\right)\cos\varphi_{b}\left(k\right)-\sin\varphi_{b}\left(k\right) & 0\\ 0 & 0 & 1 \end{array}\right],$$

(11) $$M_{2}\left(k\right)=\left[\begin{array}{ccc} -\left(h\left(k\right)+R_{e}\right)\sin\varphi_{b}\left(k\right)\cos\lambda_{b}\left(k\right) & -\left(h\left(k\right)+R_{e}\right)\cos\varphi_{b}\left(k\right)\sin\lambda_{b}\left(k\right) & \cos\varphi_{b}\left(k\right)\cos\lambda_{b}\left(k\right)\\ -\left(h\left(k\right)+R_{e}\right)\sin\varphi_{b}\left(k\right)\sin\lambda_{b}\left(k\right) & \left(h\left(k\right)+R_{e}\right)\cos\varphi_{b}\left(k\right)\cos\lambda_{b}\left(k\right) & \cos\varphi_{b}\left(k\right)\sin\lambda_{b}\left(k\right)\\ \left(h\left(k\right)+R_{e}\right)\cos\varphi_{b}\left(k\right) & 0 & \sin\varphi_{b}\left(k\right) \end{array}\right],$$

where P(k) is the pitch angle of the guide star, A(k) is the azimuth angle of the guide star, h(k) is the height of the missile, $R_{e}$ is the earth radius, $\varphi_{b}\left(k\right)$ is the latitude of the missile, and $\lambda_{b}\left(k\right)$ is the longitude of the missile. Denote $Z_{2}\left(k\right)={\left[\begin{array}{ccc} \delta P(k) & \delta A(k) & \delta h(k) \end{array}\right]}^{T}$, that is,

(12) $$Z_{2}\left(k\right)=T\left(k\right)\delta r_{l}\left(k\right)+V_{2}\left(k\right),$$

where $T\left(k\right)=M_{1}\left(k\right){\left(C_{i}^{1}C_{e}^{i}\left(k\right)M_{2}\left(k\right)\right)}^{-1}$, and $V_{2}\left(k\right)={\left[\begin{array}{ccc} v_{P}\left(k\right) & v_{A}\left(k\right) & v_{h}\left(k\right) \end{array}\right.}^{T}$.

Combined with Equations (5) and (12), the total measurement equation can be expressed:

(13) $$Z\left(k\right)=\left[\begin{array}{c} Z_{1}\left(k\right)\\ Z_{2}\left(k\right) \end{array}\right]=H\left(k\right)X\left(k\right)+V\left(k\right),$$

where the measurement matrix H(k) is:

(14) $$H\left(k\right)=\left[\begin{array}{cccc} M_{0}\left(k\right) & O_{3\;\times \;3} & O_{3\;\times \;3} & O_{3\;\times \;6}\\ O_{3\;\times \;3} & O_{3\;\times \;3} & T(k) & O_{3\;\times \;6} \end{array}\right],$$

and O is the zero matrix. The measurement noises V(k) are expressed as:

(15) $$V\left(k\right)={\left[\begin{array}{cc} V_{1}\left(k\right) & V_{2} \end{array}\left(k\right)\right]}^{T},$$

and it is independent of the state noises W(k) in Equation (3). Compared with the traditional mode, the deeply integrated mode adds the dimension of the measurements. In this way, the computational complexity will increase, which is analyzed in Section 3.3.

In many cases, V(k) denotes the Gaussian white noises [4,13,14,43,44,45]. However, in practice, due to the sensor faults or the complex environment, the measurement noises may be contaminated noises with different distributions or include large outliers. It can be shown as follows:

(16) $$V\left(k\right)=V_{k}^{\left(1\right)}+V_{k}^{\left(2\right)}$$

or

(17) $$V\left(k\right)=V_{k}^{\left(1\right)}+\Delta V_{k}$$

where $V_{k}^{\left(1\right)}$ denotes the Gaussian white noises, $V_{k}^{\left(2\right)}$ is distributed differently from $V_{k}^{\left(1\right)}$, and $\Delta V_{k}$ is the outlier.

The non-Gaussian noises can influence the performance of the navigation system; therefore, it is necessary to use the robust filter to solve the problem. The maximum correntropy Kalman filter is newly designed to effectively tackle the non-Gaussian noise.

#### 2.2.3. Observability Analysis

The observability analysis is needed to determine the efficiency of the Kalman filter designed for the purpose of estimating the system state variable [46]. If the system observability is poor, the estimation of the state will be inaccurate. Therefore, it is necessary to analyze the observability of the new integrated navigation system.

Considering the state Equation (3) and the measurement Equation (13), according to [47], the observability matrix $U_{1}$ of the deeply integrated navigation system is as follows:

(18) $$U_{1}=\left[\begin{array}{c} H\\ H\Phi \\ \vdots \\ H\Phi^{n_{X}-1} \end{array}\right]$$

where H is the measurement matrix of the deeply integrated method, Φ denotes the discrete version of the state transition matrix, $n_{x}$ is the dimension of state variable, which is 15 in the deeply integrated navigation system, as described in Equation (1), so $\mathrm{rank}\left(U_{1}\right)=n_{x}=15$. Hence, the SINS/CNS deeply integrated navigation mode is observable. For comparison, the observability matrix of the traditional navigation mode $U_{2}$ is analyzed as follows:

(19) $$U_{2}=\left[\begin{array}{c} M\\ M\Phi \\ \vdots \\ M\Phi^{n_{X}-1} \end{array}\right],$$

where M is the measurement matrix of the traditional method. Thus, $\mathrm{rank}\left(U_{2}\right)=6<n_{x}$. Therefore, the traditional integrated navigation system is unobservable. In other words, the new navigation system, compared with the traditional one, will have a more precise estimation.

## 3. The Maximum Correntropy Unscented Kalman Filter

### 3.1. Maximum Correntropy Criterion

Correntropy [48] denotes the measure of an information entropy field, which defines the novel metric in the sample space, using the information available in the higher-order statistics of the signals. Given two random variables with the joint probability density function (PDF) $p\left(a,b\right)$, the definition of correntropy is expressed as follows.

**Definition** **1.** *Given two random variables A and B, the correntropy is defined as:*

(20) $$\begin{array}{c} C_{\lambda }\left(A,B\right)=E\left[\kappa \left(A,B\right)\right]=\;\int \int \;\kappa_{\lambda }\left(a,b\right)p\left(a,b\right)dadb, \end{array}$$

*where E denotes the expectation, $\kappa_{\lambda }\left(\cdot ,\cdot \right)$ denotes a real-valued continuous, symmetric, and nonnegative definite kernel function λ>0 denotes the kernel bandwidth.*

The Gaussian kernel, which is expressed as follows, is used as the kernel function in Definition 1:

(21) $$\kappa_{\lambda }\left(a,b\right)=G_{\lambda }\left(e\right)=\exp\left(-\frac{e^{2}}{2\lambda^{2}}\right),$$

where e=a−b.

In many practical cases, there are a finite amount of samples to estimate the unknown joint PDF $p\left(a,b\right)$. Hence, the sample mean estimator can calculate the correntropy as follows:

(22) $${\hat{C}}_{\lambda }\left(A,B\right)=\frac{1}{N}\sum_{i=1}^{N}G_{\lambda }\left(e_{i}\right),$$

where

(23) $$e_{i}=a_{i}-b_{i},$$

${\left\{a_{i},b_{i}\right\}}_{i=1}^{N}$ denotes *N* samples, which are drawn from $p\left(a,b\right)$.

**Figure 3:**
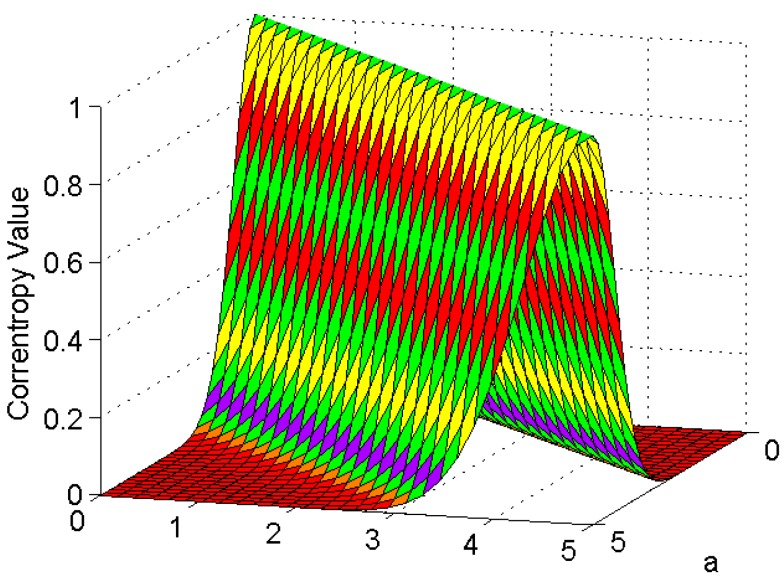
Correntropy when λ=1.

Assuming λ=0.5, $a_{i}\in \left[0,5\right]$, $b_{i}\in \left[0,5\right]$, and $p\left(a,b\right)=\frac{1}{2}e^{-\left(a^{2}\;+\;b^{2}\right)}$. The current correntropy value is depicted in Figure 3.

According to Figure 3, correntropy is considered a correlation scale in the joint space, which reaches maximum under the conditions that $a\left(i\right)=b\left(i\right)$. Based on this, the cost function can be defined to represent the maximum correntropy criterion (MCC) as follows:

(24) $$J_{MCC}=\max\sum_{i=1}^{N}G_{\lambda }\left(e_{i}\right).$$

From Figure 3, it is manifest that MCC represents a local similarity criterion that reflects the maximum error probability density from the view of probabilistic meaning [48]. Non-Gaussian noises, such as large outliers and Gaussian mixture noises, can be handled effectively on the basis of the property [49]. That is to say, MCC reaches the maximum value on the joint space’s bisector because the value of Gaussian kernel maximizes on the line A=B, which is different from the mean square error (MSE) estimation [50]. Based on this, MCC has been used for parameter estimation in recent years [34,35,51].

### 3.2. The Maximum Correntropy Unscented Kalman Filter

The MCUKF is a novel algorithm combined with a UKF framework based on the MCC, and can perform better for nonlinear systems in non-Gaussian noise environments [37].

Consider the state equation and the measurement equation described in Equations (2) and (13) in Section 2:

(25) $$\left\{\begin{array}{c} X\left(k\right)=f\left(X\left(k-1\right),W\left(k-1\right)\right)\\ Z\left(k\right)=H\left(k\right)X\left(k\right)+V\left(k\right), \end{array}\right.$$

$W\left(k-1\right)$ and $V\left(k\right)$ are mutually uncorrelated process noises and measurement noises, respectively. The mean of both noises are zero and the covariances of them are expressed as:

(26) $$E\left[W\left(k-1\right)W^{T}\left(k-1\right)\right]=Q\left(k-1\right),\;\;E\left[V\left(k\right)V^{T}\left(k\right)\right]=R\left(k\right).$$

Similar to the Kalman filter, the MCUKF includes the time update and the measurement update.

#### 3.2.1. Time Update

The sigma points set are generated from the estimated state $\hat{X}\left(k-1|k-1\right)$ and the covariance matrix $P\left(k-1|k-1\right)$ at the previous step (k−1).

(27) $$\begin{array}{c} \chi^{\left(0\right)}\left(k-1|k-1\right)=\hat{X}\left(k-1|k-1\right)\\ \chi^{\left(i\right)}\left(k-1|k-1\right)=\hat{X}\left(k-1|k-1\right)+{\left(\sqrt{\left(n_{x}+\lambda \right)P\left(k-1|k-1\right)}\right)}_{i},\;\;\;\;i=1,2,\ldots ,n_{x}\\ \chi^{\left(i\right)}\left(k-1|k-1\right)=\hat{X}\left(k-1|k-1\right)-{\left(\sqrt{\left(n_{x}+\lambda \right)P\left(k-1|k-1\right)}\right)}_{i-n},\;\;i=n_{x}+1,n_{x}+2,\ldots ,2n_{x}, \end{array}$$

where $n_{x}$ denotes the dimension number of the state, ${\left(\sqrt{\left(n_{x}+\lambda \right)P\left(k-1|k-1\right)}\right)}_{i}$ denotes the *i*-th column of $\sqrt{\left(n_{x}+\lambda \right)P\left(k-1|k-1\right)}$ , and the composite scaling factor λ is expressed as:

(28) $$\lambda \;=\;\alpha^{2}\left(n_{x}+\kappa \right)-n_{x},$$

where κ is a parameter that is set to $\left(3-n_{x}\right)$ [37], and α controls the distribution conditions of the sigma points.

The unscented transformed points are given as:

(29) $$\chi^{\left(i\right)}\left(k|k-1\right)=f\left(\chi^{\left(i\right)}\left(k-1|k-1\right),W\left(k-1\right)\right),i=0,1,\ldots ,2n_{x}.$$

The predicted state and the covariance matrix are estimated as:

(30) $$\hat{X}\left(k|k-1\right)=\sum_{i=0}^{2n_{x}}\omega_{c}^{\left(i\right)}\chi^{\left(i\right)}\left(k|k-1\right)$$

(31) $$\begin{array}{c} P\left(k|k-1\right)=\\ \sum_{i=0}^{2n_{x}}\omega_{c}^{\left(i\right)}\left[\hat{X}\left(k|k-1\right)-\chi^{\left(i\right)}\left(k|k-1\right)\right]{\left[\hat{X}\left(k|k-1\right)-\chi^{\left(i\right)}\left(k|k-1\right)\right]}^{T}+Q\left(k-1\right) \end{array}$$

where the weights of the sigma points are as follows:

(32) $$\left\{\begin{array}{c} \omega_{m}^{\left(0\right)}=\frac{\lambda }{n+\lambda }\\ \omega_{c}^{\left(0\right)}=\frac{\lambda }{n+\lambda }+\left(1-\alpha^{2}+\beta \right)\\ \omega_{m}^{\left(i\right)}=\omega_{c}^{\left(i\right)}=\frac{\lambda }{2(n+\lambda )},i=1,2,\ldots ,2n_{x}, \end{array}\right.$$

where α is expressed in Equation (28) and β is a weighted parameter that is non-negative. When the measurement data are not avaliable, $\hat{X}\left(k|k-1\right)$ can replace the current state estimation.

The sigma points of the prior state mean and the predicted covariance are set as:

(33) $$\begin{array}{c} \chi^{\left(0\right)}\left(k|k-1\right)=\hat{X}\left(k|k-1\right)\\ \chi^{\left(i\right)}\left(k|k-1\right)=\hat{X}\left(k|k-1\right)+{\left(\sqrt{\left(n_{x}+\lambda \right)P\left(k|k-1\right)}\right)}_{i},i=1,2,\ldots ,n_{x}\\ \chi^{\left(i\right)}\left(k|k-1\right)=\hat{X}\left(k|k-1\right)-{\left(\sqrt{\left(n_{x}+\lambda \right)P\left(k|k-1\right)}\right)}_{i-n},i=n_{x}+1,n_{x}+2,\ldots ,2n_{x}. \end{array}$$

The sigma points are then transferred using the measurement equation as:

(34) $$Z^{\left(i\right)}\left(k|k-1\right)=H\left(k\right)\chi^{\left(i\right)}\left(k|k-1\right),i=0,1,\ldots ,2n_{x}.$$

The predicted measurement mean can be estimated as:

(35) $$\hat{Z}\left(k|k-1\right)=\sum_{i=1}^{2n_{x}}\omega_{m}^{i}Z^{\left(i\right)}\left(k|k-1\right).$$

#### 3.2.2. Measurement Update

Next, the measurement update is calculated based on the MCC. For the model described in the previous section, we have

(36) $$\left[\begin{array}{c} \hat{X}\left(k|k-1\right)\\ Z\left(k\right) \end{array}\right]=\left[\begin{array}{c} I\\ H\left(k\right) \end{array}\right]X\left(k\right)+u\left(k\right),$$

where I denotes the unit matrix, and $u\left(k\right)=\left[\begin{array}{c} \hat{X}\left(k|k-1\right)-X\left(k\right)\\ V\left(k\right) \end{array}\right]$ , with

(37) $$\begin{array}{c} E\left[u\left(k\right)u^{T}\left(k\right)\right]\\ =\left[\begin{array}{cc} P\left(k|k-1\right) & O\\ O & R\left(k\right) \end{array}\right]\\ =\left[\begin{array}{cc} B_{p}\left(k|k-1\right)B_{p}^{T}\left(k|k-1\right) & O\\ O & B_{r}\left(k\right)B_{r}^{T}\left(k\right) \end{array}\right]\\ =B\left(k\right)B^{T}\left(k\right), \end{array}$$

where $B\left(k\right)$, $B_{p}\left(k|k-1\right)$, and $B_{r}\left(k\right)$ is calculated on the basis of Cholesky decomposition of $E\left[u\left(k\right)u^{T}\left(k\right)\right]$, the predicted covariance $P\left(k|k-1\right)$ , and the covariance of the measurement noises $R\left(k\right)$, respectively. In fact, u denotes a random variable matrix, which consists of the prior estimation error of the state and the measurements prediction error. Equation (36) is multiplied by $B^{-1}\left(k\right)$ on the left of both sides, and we get

(38) $$D\left(k\right)=w\left(k\right)X\left(k\right)+e\left(k\right),$$

where $D\left(k\right)=B^{-1}\left(k\right)\left[\begin{array}{c} \hat{X}\left(k|k-1\right)\\ Z\left(k\right) \end{array}\right]$ , $w\left(k\right)=B^{-1}\left(k\right)\left[\begin{array}{c} I\\ H\left(k\right) \end{array}\right]$ , and $e\left(k\right)=B^{-1}\left(k\right)u\left(k\right)$. In this way, $e\left(k\right)$ denotes a new random variable matrix, which is just multiplied by a matrix $B^{-1}$.

Now the MCC for Equation (24) based on the cost function can be rewritten as follows:

(39) $$J_{MCC}\left(X\left(k\right)\right)=\frac{1}{N}\sum_{i=1}^{N}G\left(e_{i}\left(k\right)\right)=\frac{1}{N}\sum_{i=1}^{N}G\left(d_{i}\left(k\right)-w_{i}\left(k\right)X\left(k\right)\right),$$

where $e_{i}\left(k\right)$ denotes the *i*-th unit of $e\left(k\right)$, $d_{i}\left(k\right)$ denotes the *i*-th unit of $D\left(k\right)$, $w_{i}\left(k\right)$ denotes the *i*-th row of $w\left(k\right)$, and $N=n_{x}+n_{z}$ denotes the entire dimension of $D\left(k\right)$, which represents the sum of the dimension of state variable $X\left(k\right)$ and the measurement $Z\left(k\right)$. In fact, $d_{i}\left(k\right)$ is equivalent to $a_{i}$ in Equation (23), and $w_{i}\left(k\right)X\left(k\right)$ is equivalent to $b_{i}$ in Equation (23). That is, $e_{i}\left(k\right)$ is equivalent to $e_{i}$ in Equations (22) and (23), and $J_{MCC}\left(X\left(k\right)\right)$ is equivalent to ${\hat{C}}_{\lambda }\left(A,B\right)$ in Equation (22).

In particular, according to Figure 3, when the error $e_{i}\left(k\right)$ reaches the minimum, the correntropy reaches its maximum value. In other words, the optimal estimation of the state can minimize the error and maximize the correntropy as well. In this way, the optimal estimate of $X\left(k\right)$ is considered with respect to MCC.

Then, the optimal estimate under the MCC criterion of $X\left(k\right)$ is

(40) $$\hat{X}\left(k\right)=\arg\max_{X}J_{MCC}\left(X(k)\right)=\arg\max_{X(k)}\sum_{i=1}^{N}G_{\lambda }\left(e_{i}\left(k\right)\right).$$

The optimal solution can thus be obtained by solving

(41) $$\frac{\partial J_{MCC}\left(X(k)\right)}{\partial X\left(k\right)}=0.$$

It follows that

(42) $$\begin{array}{c} X\left(k\right)={\left(\sum_{i=1}^{N}\left[G_{\lambda }\left(e_{i}\left(k\right)\right)w_{i}^{T}\left(k\right)w_{i}\left(k\right)\right]\right)}^{-1}\times \\ \;\;\;\;\;\;\;\;\;\;\;\;\;\;\;\;\;\;\;\;\;\;\;\;\;\;\;\;\;\;\;\;\;\;\;\;\;\left(\sum_{i=1}^{N}\left[G_{\lambda }\left(e_{i}\left(k\right)\right)w_{i}^{T}\left(k\right)d_{i}\left(k\right)\right]\right). \end{array}$$

The optimal solution expressed by Equation (42) is actually a fixed-point equation of $X\left(k\right)$ and can be rewritten as:

(43) $$X\left(k\right)=g\left(X\left(k\right)\right),$$

with

$$\begin{array}{c} g\left(X\left(k\right)\right)={\left(\sum_{i=1}^{N}\left[G_{\lambda }\left(d_{i}\left(k\right)-w_{i}\left(k\right)X\left(k\right)\right)w_{i}^{T}\left(k\right)w_{i}\left(k\right)\right]\right)}^{-1}\times \\ \;\;\;\;\;\;\;\;\;\;\;\;\;\;\;\;\;\;\;\;\;\;\;\;\;\;\;\;\;\;\;\;\;\;\;\;\;\;\;\;\;\;\;\;\;\;\;\;\;\;\;\;\;\;\;\;\left(\sum_{i=1}^{N}\left[G_{\lambda }\left(d_{i}\left(k\right)-w_{i}\left(k\right)X\left(k\right)\right)w_{i}^{T}\left(k\right)d_{i}\left(k\right)\right]\right). \end{array}$$

A fixed-point iterative algorithm can be readily obtained as [51]:

(44) $$\hat{X}{\left(k\right)}_{t^{*}+1}=g\left(\hat{X}{\left(k\right)}_{t^{*}}\right)$$

where $\hat{X}{\left(k\right)}_{t^{*}}$ denotes the solution at the fixed-point iteration $t^{*}$.

The fixed-point Equation (42) can also be expressed in matrix form as:

(45) $$X\left(k\right)={\left(w^{T}\left(k\right)C\left(k\right)w\left(k\right)\right)}^{-1}w^{T}\left(k\right)C\left(k\right)D\left(k\right),$$

where $C\left(k\right)=\left[\begin{array}{cc} C_{X}\left(k\right) & O\\ O & C_{Z}\left(k\right) \end{array}\right]$ , and

$$C_{X}\left(k\right)=diag\left(G_{\lambda }\left(e_{1}\left(k\right)\right),\ldots ,G_{\lambda }\left(e_{n_{x}}\left(k\right)\right)\right)$$

$$C_{Z}\left(k\right)=diag\left(G_{\lambda }\left(e_{n_{x}+1}\left(k\right)\right),\ldots ,G_{\lambda }\left(e_{n_{x}+n_{z}}\left(k\right)\right)\right).$$

Equation (45) can be further expressed as follows (the detailed derivation is in [52]):

(46) $$X\left(k\right)=\hat{X}\left(k|k-1\right)+\bar{K}\left(k\right)\left(Z\left(k\right)-\hat{Z}\left(k|k-1\right)\right)$$

(47) $$\left\{\begin{array}{c} \bar{K}\left(k\right)=\bar{P}\left(k|k-1\right)H^{T}\left(k\right){\left(H\left(k\right)\bar{P}\left(k|k-1\right)H^{T}\left(k\right)+\bar{R}\left(k\right)\right)}^{-1}\\ \bar{P}\left(k|k-1\right)=B_{p}\left(k|k-1\right)C_{X}^{-1}\left(k\right)B_{p}^{T}\left(k|k-1\right)\\ \bar{R}\left(k\right)=B_{r}\left(k\right)C_{Z}^{-1}\left(k\right)B_{r}^{T}\left(k\right) \end{array}\right..$$

With the above derivations, we summarize the proposed MCUKF algorithm as follows:

Step 1: Choose a proper kernel bandwidth λ and a small positive number ε; the estimate $\hat{X}\left(0|0\right)$ and the covariance matrix $P\left(0|0\right)$ are initialized; let k=1.
Step 2: Use Equations (27)–(32) to obtain $\hat{X}\left(k|k-1\right)$ and $P\left(k|k-1\right)$, and make use of Cholesky decomposition to calculate $B_{p}\left(k|k-1\right)$.
Step 3: According to Equations (32)–(35), compute the predicted measurement $\hat{Z}\left(k|k-1\right)$ and use Equations (25) and (30) to construct the model (36).
Step 4: Transform Equation (36) in to Equation (38). Let $t^{*}=1$ and $\hat{X}{\left(k|k\right)}_{0}=\hat{X}\left(k|k-1\right)$, where $\hat{X}{\left(k|k\right)}_{t^{*}}$ denotes the estimated state at the fixed-point iteration $t^{*}$.
Step 5: Use Equations (48)–(54) to compute $\hat{X}{\left(k|k\right)}_{t^{*}}$:
  (48) $$\hat{X}{\left(k|k\right)}_{t^{*}}=\hat{X}\left(k|k-1\right)+\tilde{K}\left(k\right)\hat{Z}\left(k|k-1\right)$$
  and
  (49) $$\tilde{K}\left(k\right)=\tilde{P}\left(k|k-1\right)H^{T}\left(k\right){\left(H\left(k\right)\tilde{P}\left(k|k-1\right)H^{T}\left(k\right)+\tilde{R}\left(k\right)\right)}^{-1}$$
  (50) $$\tilde{P}\left(k|k-1\right)=B_{p}\left(k|k-1\right){\tilde{C}}_{X}^{-1}\left(k\right)B_{p}^{T}\left(k|k-1\right)$$
  (51) $$\tilde{R}\left(k\right)=B_{r}\left(k\right){\tilde{C}}_{Z}^{-1}\left(k\right)B_{r}^{T}\left(k\right)$$
  (52) $${\tilde{C}}_{X}\left(k\right)=diag\left(G_{\lambda }\left({\tilde{e}}_{1}\left(k\right)\right),\ldots ,G_{\lambda }\left({\tilde{e}}_{n_{x}}\left(k\right)\right)\right)$$
  (53) $${\tilde{C}}_{Z}\left(k\right)=diag\left(G_{\lambda }\left({\tilde{e}}_{n_{x}+1}\left(k\right)\right),\ldots ,G_{\lambda }\left({\tilde{e}}_{n_{x}+n_{z}}\left(k\right)\right)\right)$$
  (54) $${\tilde{e}}_{i}\left(k\right)=d_{i}\left(k\right)-w_{i}\left(k\right)\hat{X}{\left(k|k\right)}_{t^{*}-1}.$$
Step 6: Compare the estimation of the current step and the estimation of the last step. If Equation (55) holds, set $\hat{X}\left(k|k\right)=\hat{X}{\left(k|k\right)}_{t^{*}}$ and continue to Step 6. Otherwise, go back to Step 4.
  (55) $$\frac{∥\hat{X}{\left(k|k\right)}_{t^{*}}-\hat{X}{\left(k|k\right)}_{t^{*}-1}∥}{∥\hat{X}{\left(k|k\right)}_{t^{*}-1}∥}\leq \varepsilon .$$
Step 7: Update the estimation covariance matrix, make k+1→k, and go back to Step 2.
  (56) $$P\left(k|k\right)=\left(I-\tilde{K}\left(k\right)H\left(k\right)\right)P\left(k|k-1\right){\left(I-\tilde{K}\left(k\right)H\left(k\right)\right)}^{T}+\tilde{K}\left(k\right)R\left(k\right){\tilde{K}}^{T}\left(k\right).$$

For obtaining the optimal solution in Equation (42), the MCUKF updates the state estimation through a fixed-point algorithm, which is shown as Equation (55). Therefore, the small positive number ε provides a threshold for the fixed-point iteration. When the result of the iteration is in the threshold range, we call it convergence and the solution is optimal. The convergence of the solution will be fast because of the predicted state $\hat{X}\left(k|k-1\right)$ [52].

### 3.3. Computational Complexity

For the future implementation on real hardware, it is necessary to analyze the computational complexity of the proposed approach in the article. In terms of the floating points operations, the computation complexity of the basic equations of the MCUKF is analyzed in Table 1.

The traditional unscented Kalman filter is analyzed in [53]. The computation complexity of it is

(57) $$S_{\mathrm{UKF}}=\frac{26}{3}n^{3}+15n^{2}+10n^{2}m+5n+8nm^{2}+6mn+m^{3}+3m^{2}+3m.$$

In fact, Equations (27)–(35) in the MCUKF are the same as that in the UKF. Equations (48)–(56) in the MCUKF mainly involves the different equations expressed by Equations (48)–(56). Equation (55) gives a fixed-point algorithm to update the posterior estimate of the state that can provide a stop condition in several steps. Assuming that the fixed-point iteration number is *T*, then the computation complexity of MCUKF is

(58) $$\begin{array}{c} S_{\mathrm{MCUKF}}=\left(\frac{38}{3}\;+\;2\right)Tn^{3}+\left(11\;+\;2T\right)n^{2}+\left(10+4T\right)n^{2}m+\left(2T+3\right)n\\ +\left(4T+2\right)nm^{2}+\left(1+3T\right)mn+2Tm^{3}+m \end{array}.$$

According to Equations (57) and (58), the computational complexity of the MCUKF is much higher than that of the UKF because of the fixed-point algorithm and more matrix inverse problems.

According to the complexity analysis above, the increasing dimension of the measurements of the deeply integrated mode has a higher computational complexity than the traditional mode.

### 3.4. Robustness Analysis

The robustness is a very important feature for the navigation filter algorithm, especially in the complex space environment. One of the most significant advantages of MCC is robustness that can tackle non-Gaussian noises. In fact, it uses a method similar to the Huber-based filter (HF) to improve the robustness [54]. Both of them use the weighted function to tackle the non-Gaussian noises. HF is a classical method to tackle the non-Gaussian noises, and it has been proved in detail [55]. Additionally, some articles have demonstrated the ability of MCC in accommodating non-Gaussian noises, especially outliers and Gaussian mixture noises [56,57]. Next, the algorithm robustness is compared with HF through the weighting function.

HF is created based on the concept of the robustness through the maximum likelihood method. Assume that the maximum likelihood estimator (MLE) stems from the data observation $Z=\left\{z_{1},\ldots ,z_{N}\right\}$ and the state variable $X=\left\{x_{1},\ldots ,x_{N}\right\}$. Then e=Z−HX is drawn from the distribution $p\left(e;X\right)$, which is known apart from the parameters X. In this way, the MLE of X can be defined as:

(59) $$\begin{array}{c} {\hat{X}}_{MLE}=\arg\max_{X}p\left(e_{1},\ldots ,e_{N};X\right). \end{array}$$

Assuming that $e_{1},\ldots ,e_{N}$ are i.i.d. observations, then it can be rewritten as:

(60) $$\begin{array}{c} \begin{array}{cc} {\hat{X}}_{MLE} & =\arg\max_{X}\prod_{i=1}^{N}p\left(e_{i};X\right)=\arg\max_{X}\sum_{i=1}^{N}\ln p\left(e_{i};X\right)\\ & =\arg\min_{X}\sum_{i=1}^{N}\left(-\ln p\left(e_{i};X\right)\right) \end{array}. \end{array}$$

Let $\rho \left(e_{i};X\right)=a\left(-\ln p\left(e_{i};X\right)\right)+b$, where a>0 and *b* is uncorrelated with X. Thus, ${\hat{X}}_{MLE}$ obeys

(61) $$\begin{array}{c} \sum_{i=1}^{N}\rho \left(e_{i};X\right)=\sum_{i=1}^{N}\rho \left(z_{i}-Hx_{i}\right)=\min_{X}\sum_{i=1}^{N}\rho \left(e_{i};X\right). \end{array}$$

In fact, the errors in the target track model are always considered as pure Gaussian white noises with zero mean and constant variance $\sigma^{2}$ in some parametric models. In these cases, the maximum likelihood estimation can be solved. However, in practice, the external environment influences the measurement equipment and causes the filter algorithm to break down. Hence, the non-Gaussian noises in the measurements cannot be avoided. A typical non-Gaussian noises are Gaussian mixture noises, which obey the Gaussian distributions with different mean values and variances with most errors. For instance, assume that the distribution is expressed as:

(62) $$\begin{array}{c} F\left(e_{i};X\right)=\left(1-\upsilon \right)F_{0}\left(e_{i};X\right)+\upsilon F_{c}\left(e_{i};X\right), \end{array}$$

where $F\left(\cdot \right)$, $F_{0}\left(\cdot \right)$, and $F_{c}\left(\cdot \right)$ denote the Gaussian mixture distribution, the known normal Gaussian distribution, and an unknown contaminated distribution, respectively, and υ is the contamination ratio. It is much smaller than 1. For Equation (62), it is of necessity to solve the maximum likelihood estimation through Equation (61).

For the purpose of solving the problem, Huber proposed that ρ can change freely within limits, leading to the birth of *M*-estimation, which is an effective way of solving Equation (61).

*M*-estimator is defined as a generalization of MLE as:

(63) $$\begin{array}{c} \min_{X}\sum_{i=1}^{N}\rho \left(e_{i};X\right)\;or\;\sum_{i=1}^{N}\varphi \left(e_{i};X\right)=0\;with\;\varphi \left(e_{i};X\right)=\frac{d\rho \left(e_{i};X\right)}{de_{i}}, \end{array}$$

where $\rho \left(e\right)$ denotes the cost or penalty function.

Let

(64) ρMCCei=1−exp−ei2/2λ21−exp−ei2/2λ22π2πλ

where λ denotes the kernel bandwidth.

Then

(65) min∑i=1NρMCCei=min∑i=1N1−exp−ei2/2λ21−exp−ei2/2λ22π2πλ⇔max∑i=1Nexp−ei2/2λ2exp−ei2/2λ22π2πλ=max∑i=1NGλei.

Therefore, MCC is equivalent to Equation (64):

$$\min_{X}\;\sum_{i=1}^{N}\rho_{MCC}\left(e_{i}\right)\;is\;equivalent\;to\;MCC=\max_{X}\;\sum_{i=1}^{N}G_{\lambda }\left(e_{i}\right).$$

According to the *M*-estimation problem, $\psi_{MCC}\left(e_{i}\right)$, as well as the weighting function of MCC, can be rewritten as:

(66) $$\begin{array}{c} \psi_{MCC}\left(e_{i}\right)=\frac{{\rho_{MCC}}^{\prime }\left(e_{i}\right)}{e_{i}}, \end{array}$$

where ${\rho_{MCC}}^{\prime }\left(e_{i}\right)=\frac{d\rho_{MCC}\left(e_{i}\right)}{de_{i}}$. Therefore,

(67) ψMCCei=exp−ei2/2λ2exp−ei2/2λ22π2πλ3.

On the basis of analysis of the weighting function above, we will compare it with the Huber filter to prove the superiority of MCC.

The weighted functions of them are shown in Table 2.

**Figure 4:**
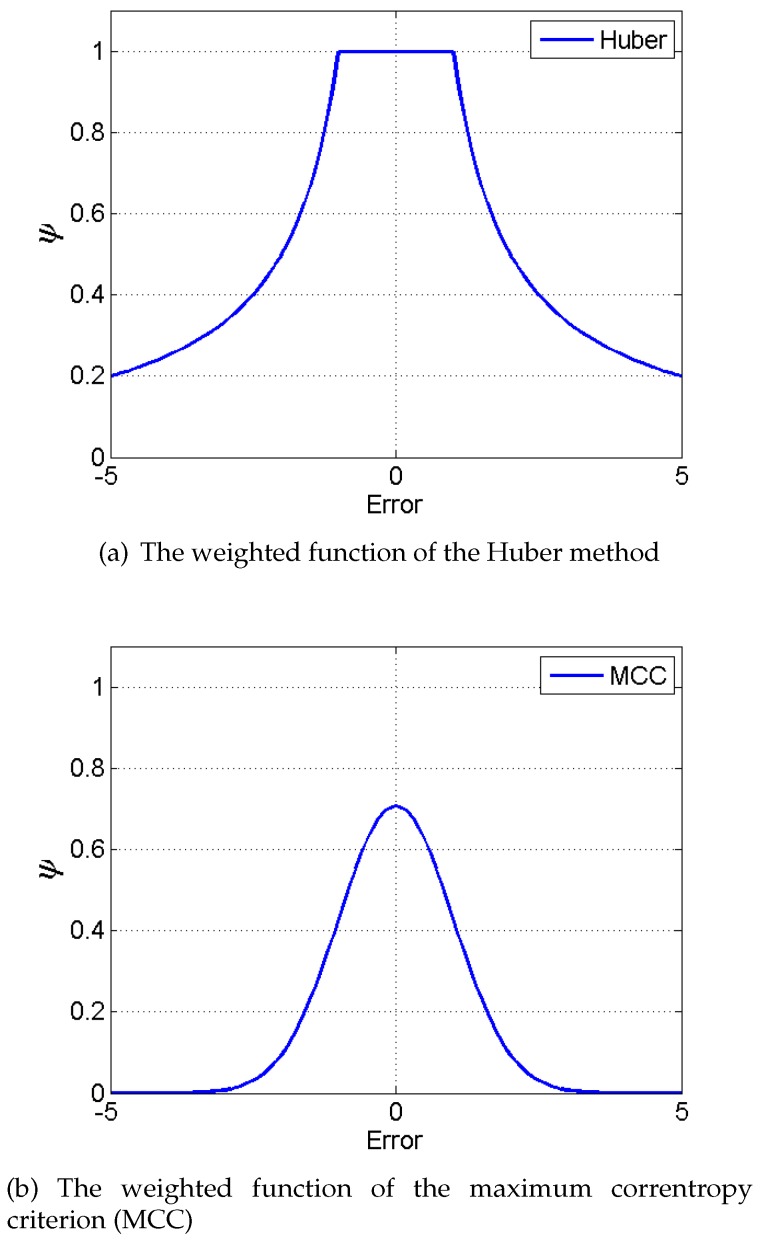
The weight comparison with the error growth of two methods where λ=δ=1.

Some researchers have performed robustness analysis of MCC in theory. The robustness of two methods are shown through Figure 4 to intuitively analyze the robustness of MCC.

In Figure 4, the weight of HF decreases rapidly as the estimate residual grows. However, the weight of MCC approaches zero faster compared with that of the Huber method. In addition, with the growth of the error, the weight of the Huber method is not zero. In other words, Huber-based methods can select more measurements containing large errors than MCC-based methods, which may cause large errors to the filter algorithm, although with small weights. Therefore, the filter based on the MCC is more robust than the Huber-based filter.

## 4. Simulation Results

In this section, the superiority of SINS/CNS deeply integrated navigation is proved firstly. The performances of the MCUKF are then illustrated under the conditions of the Gaussian noises, the large outliers, and the Gaussian mixture noises, respectively. The estimation performance are compared on the basis of the following indices:

(68) $$\mathrm{Residual}\left(k\right)=X\left(k\right)-\hat{X}\left(k|k\right),\;\;k=1,\ldots ,m,$$

(69) $$\mathrm{RMSE}=\sum_{j=1}^{L}\sqrt{\frac{1}{m}\sum_{i=1}^{m}{\left(X\left(k\right)-\hat{X}\left(k|k\right)\right)}^{2}}/L,j=1,\ldots ,M,$$

where *m* is the simulation time steps and *L* represents the total simulation numbers. In addition, the time cost of every method is given to compare the computational complexity. All methods are implemented in the same precision (64-point floating point) in MATLAB running on a PC with processor Intel(R) Core(TM) i7-7500U CPU 2.70GHz and with 8 GB of installed memory (RAM).

The simulation parameters are as Table 3.

**Figure 5:**
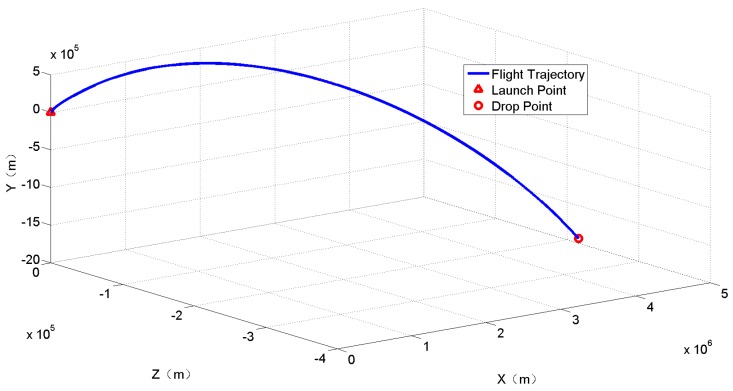
Missile flight trajectory.

The missile trajectory is shown in Figure 5.

**Figure 6:**
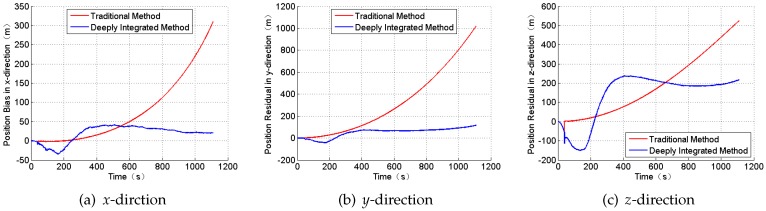
The position residual.

**Figure 7:**
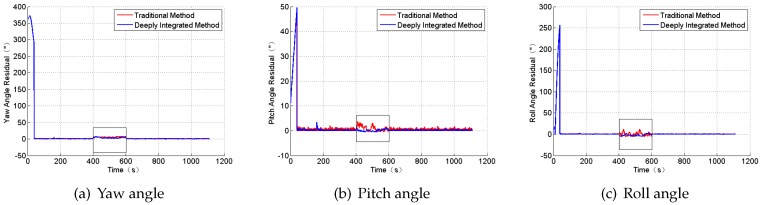
The attitude angle residual.

To prove the high performance of the new integrated navigation system, a simulation is done only under the condition of the Gaussian white noises. Compared with the traditional integrated mode, the position and the attitude angle residual are shown in Figure 6 and Figure 7.

The RMSE of the position and attitude angle and the time cost of every filter are shown in Table 4.

From the Figures and the table above, the navigation position error has evidently improved when constructing the measurement $Z_{2}$ in Equation (12). In addition, the SINS/CNS deeply integrated navigation mode for the ballistic missile can tackle the divergence of the position error effectively, which obeys the observability analysis results. However, the newly proposed navigation system needs more time to gain the results.

Next, we will compare the MCUKF performance with other algorithms under the conditions of the Gaussian noises, the large outliers, and the Gaussian mixture noises for SINS/CNS deeply integrated navigation.

### 4.1. Case 1: Gaussian Noises

**Figure 8:**
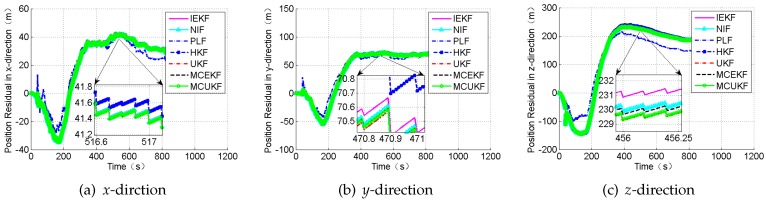
The position residual under the condition of Gaussian noise.

**Figure 9:**
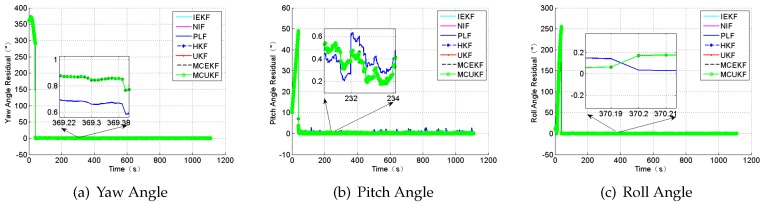
The attitude angle residual under the condition of Gaussian noise.

In this section, we will apply the MCUKF on SINS/CNS deeply integrated navigation under the conditions of the Gaussian noises to compare performances with other filter algorithms including the HKF [55], the IEKF, the NIF, the PLF, the UKF, and the MCEKF [36]. The simulation parameters are set as Table 1. The simulation results are shown in Figure 8 and Figure 9, and Table 5.

According to Table 5, PLF demonstrates the highest accuracy among the seven filters in this condition because of the a posteriori estimation and the iteration. It can be observed that robust filters cannot always perform as well as the PLF, the NIF, or the UKF under the condition of the Gaussian noises.

### 4.2. Case 2: Large Outliers

**Figure 10:**
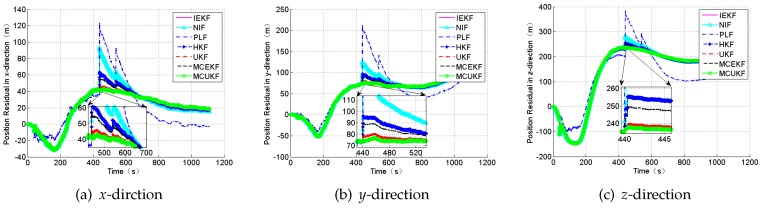
The position residual under the condition of the large outliers.

**Figure 11:**
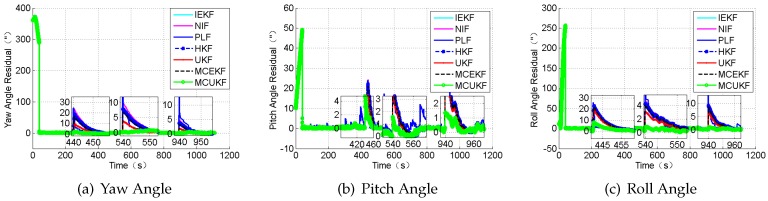
The attitude angle residual under the condition of the large outliers.

In this section, the case in which the measurement value including large outliers is considered. The attitude outliers are added artificially to the attitude angle data at the 5000^*th*^ and 9000^*th*^ epochs, respectively. The position outliers are also added artificially to the position data at the 5000^*th*^ epochs. The constant outliers are added to all epochs between the 4001^*th*^ to the 4005^*th*^ to the attitude angle data. The constant outliers are added to all epochs between 4001^*th*^ and the 4005^*th*^ to the position data. The obtained position errors and the attitude angle errors are displayed in Figure 10 and Figure 11, and Table 6.

According to the presented results, it can be concluded that, in the presence of outliers, the filter results mostly depend on the outliers. In addition, Figure 10 and Figure 11 show that the HKF, the MCEKF, and the MCUKF can resist the influence of the outliers to some extent. Furthermore, the two figures show that the MCUKF has better performance than the other algorithms, so the influence of the single and the constant outliers is reduced using the MCUKF algorithm. The RMSE values and time cost of all used filters are presented in Table 6.

Compared with other filters, the filtering algorithm of the MCEKF and the MCUKF is improved to a certain degree, especially for the outliers. The MCUKF has better performance than other filters and lower time cost than the iterated filters.

### 4.3. Case 3: Gaussian Mixture Noises

In this section, the case in which the measurement noises are the Gaussian mixture noises, whose distributions are as follows:

Star Sensor Noise: $v_{s}∼0.9N\left(0,{\left(3^{\prime \prime }\right)}^{2}\right)+0.1N\left(0,{\left(30^{\prime \prime }\right)}^{2}\right)$.

Altimeter Noise: $v_{h}∼0.9N\left(0,{\left(50m\right)}^{2}\right)+0.1N\left(0,{\left(500m\right)}^{2}\right)$.

**Figure 12:**
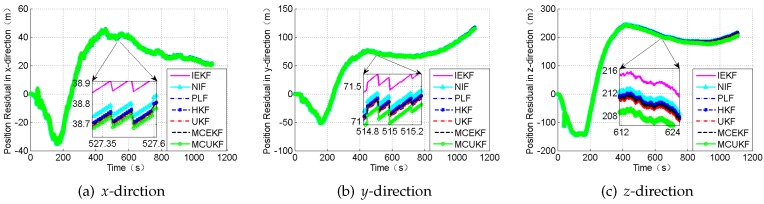
The position residual under the condition of the Gaussian mixture noises.

**Figure 13:**
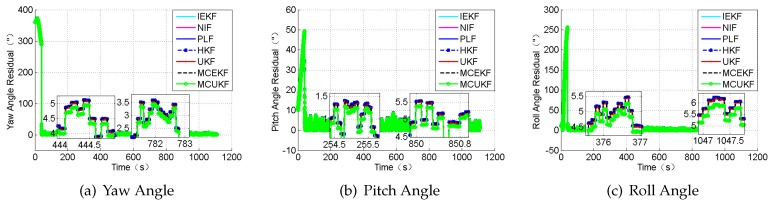
The attitude angle residual under the condition of the Gaussian mixture noises.

The obtained position errors and the attitude angle errors are displayed in Figure 12 and Figure 13, and Table 7.

Figure 12 and Figure 13 reveal the residual using different filters under the conditions of the Gaussian mixture noises. In addition, Table 7 illustrates the RMSE of the position, the attitude angle, and the time cost of every method. It can be observed that the MCUKF achieves better performance than other filters.

## 5. Conclusions

SINS/CNS deeply integrated navigation, which consists in modified SINS/CNS navigation, is introduced, and the observability of the navigation system was analyzed. However, the simulation results demonstrate that the new system needs more time. Measurement noises are often the research focus with regard to navigation systems. The non-Gaussian noises, including the large outliers and the Gaussian mixture noises, always exist during the measurement process. On the basis of the maximum correntropy theory, the maximum correntropy unscented Kalman filter (MCUKF) is a newly designed robust unscented Kalman filter (UKF). The robustness for state estimation, which can resist the effects of non-Gaussian noises effectively, is also analyzed and compared with a Huber-based filter. Simulated experimental results demonstrate that the MCC-based filter provides much better performance when tackling the non-Gaussian noises of SINS/CNS deeply integrated navigation, but with a higher computational complexity than the traditional UKF.

In practical applications, the added star sensors and the altimeter of the deeply integrated system will increase the load of the missile. Future work will focus on the installation method of the measurement facilities and applications in real scenarios. The coupled problem of multiple sensors may also need to be solved.

## Figures and Tables

**Table 1:** Computational complexities of some equations, where *n* denotes the dimension of the state variable, *m* denotes the dimension of the measurements.

Equation	Addition/Subtraction and Multiplication	Equation	Addition/Subtraction and Multiplication
(25)	$\frac{1}{3}n^{3}+3n^{2}$	(47)	$4n^{2}m+4nm^{2}-3nm$
(27)	$4n^{3}-n$	(48)	$2n^{3}$
(28)	$2n^{2}\;+\;2n$	(49)	$2m^{3}$
(29)	$4n^{3}+5n^{2}+2n$	(50)	$2n^{2}$
(31)	$\frac{1}{3}n^{3}+3n^{2}$	(51)	2nm
(32)	$4n^{2}m-m$	(52)	2n
(33)	2nm+2m	(54)	$4n^{3}+6n^{2}m-2n^{2}\;+\;2nm^{2}-nm$
(46)	4mn		

**Table 2:** The weighted functions of two methods.

Item	Penalty Function	Weighted Function
Huber	$\rho_{\mathrm{Huber}}\left(e_{i}\right)=\left\{\begin{array}{cc} e_{i}^{2}/2 & \left|e_{i}\right|\leq \delta \\ \delta \left(\left|e_{i}\right|-\delta \right) & \left|e_{i}\right|>\delta \end{array}\right.$	$\psi_{\mathrm{Huber}}\left(e_{i}\right)=\left\{\begin{array}{cc} 1 & \left|e_{i}\right|\leq \delta \\ \delta /\left|e_{i}\right| & \left|e_{i}\right|>\delta \end{array}\right.$
MCC	ρMCCei=1−exp−ei2/2λ21−exp−ei2/2λ22π2πλ	ψMCCei=exp−ei2/2λ2exp−ei2/2λ22π2πλ3

**Table 3:** Simulation conditions.

Parameter	Values	Parameter	Values
Initial latitude	39.98${}^{\circ }$	Random noise of altimeter	50 m
Initial longitude	116.34${}^{\circ }$	Random noise of star sensor	$3^{\prime \prime }$
Initial velocity	$\begin{array}{c} v_{x}=355.49\;m/s\\ v_{y}=v_{z}=0 \end{array}$	Time of the vertical rise	10 s
Initial pitch angle (Launching angle)	90${}^{\circ }$	Filter start time	40 s
Gravity acceleration	$g_{0}=9.78\;m/s^{2}$	Ending time of the powered phase turn	60 s
Constant drifts of the gyro	$\begin{array}{c} \varepsilon_{x}=\varepsilon_{y}=\\ \varepsilon_{z}=1^{\circ }/h \end{array}$	Time of the engine shutting off	160 s
Random noise of the gyro	$\begin{array}{c} \varepsilon_{sx}=\varepsilon_{sy}=\\ \varepsilon_{sz}=0.5^{\circ }/h \end{array}$	Total flying time	1110 s
Constant biases of the accelerometers	$\begin{array}{c} \nabla_{x}=\nabla_{y}=\\ \nabla_{z}=100\;\mathrm{ug} \end{array}$	Filter period	1 s
Random noise of the accelerometers	$\begin{array}{c} \nabla_{sx}=\nabla_{sy}=\\ \nabla_{sz}=50\;\mathrm{ug} \end{array}$	Gyro Sample period	0.01 s
Monte Carlo times	*L* =10	Star Sensor Sampling period	1 s

**Table 4:** RMSE of the position, attitude, and time cost of the two navigation methods.

	Position (m)			Attitude (${}^{\prime \prime }$)			Time (min)
	*x*	*y*	*z*	Yaw	Pitch	Roll	
Traditional Method	114.4777	434.0437	245.1516	27.4501	65.9759	6.1114	0.738
Deeply Integrated Mode	30.9459	67.5309	184.4883	27.4490	65.9718	6.1009	0.934

**Table 5:** RMSE of position, attitude, and time cost of different methods in the presence of Gaussian noise for SINS/CNS deeply integrated navigation.

	Position (m)			Attitude (${}^{\prime \prime }$)			Time (min)
	*x*	*y*	*z*	Yaw	Pitch	Roll	
IEKF	29.5693	66.0529	184.5749	27.4483	66.0447	6.0555	0.9748
NIF	29.5635	66.0312	184.2973	27.4483	66.0447	6.0555	1.3277
PLF	25.5944	66.6029	172.7334	27.4425	66.0406	6.0086	1.4536
HKF	29.6274	66.2252	186.4335	27.4483	66.0447	6.0555	0.5878
UKF	29.5598	66.0247	184.2777	27.4483	66.0447	6.0555	0.1124
MCEKF	29.5841	66.0968	185.0428	27.4483	66.0447	6.0555	0.2280
MCUKF	29.5659	66.0427	184.4676	27.4483	66.0447	6.0555	0.3230

**Table 6:** RMSE of position, attitude, and time cost of different methods in the presence of large outliers for SINS/CNS deeply integrated navigation.

	Position (m)			Attitude (${}^{\prime \prime }$)			Time (min)
	*x*	*y*	*z*	Yaw	Pitch	Roll	
	*x*	*y*	*z*	*p*	*ph*	*g*	time
IEKF	34.9042	70.5450	186.9381	27.5917	66.1098	6.2019	0.4629
NIF	34.8999	70.5382	186.9179	27.5918	66.1098	6.2021	0.8702
PLF	36.1497	73.6876	176.1497	27.5687	66.1091	6.1272	0.9432
HKF	31.5010	68.6713	185.1142	27.5874	66.1095	6.1775	0.2228
UKF	29.6882	67.6938	184.3485	27.5715	66.1088	6.1275	0.1332
MCEKF	30.8100	68.2925	184.7478	27.5852	66.1093	6.1657	0.2257
MCUKF	29.3169	67.5903	184.3414	27.5579	66.1081	6.0574	0.2665

**Table 7:** RMSE of position, attitude, and time cost of different message under the condition of the Gaussian mixture noises for SINS/CNS deeply integrated navigation.

	Position (m)			Attitude (${}^{\prime \prime }$)			Time (min)
	*x*	*y*	*z*	Yaw	Pitch	Roll	
	*x*	*y*	*z*	*p*	*ph*	*g*	time
IEKF	30.1388	67.7275	185.7022	26.6707	66.0896	6.6902	0.8293
NIF	30.0166	67.4352	183.6012	27.8260	67.2466	6.6826	1.2689
PLF	30.6610	67.5520	183.6714	27.6757	66.0895	6.6811	2.9075
HKF	30.0526	67.5224	184.2160	27.6750	66.0893	6.6787	1.3422
UKF	30.0090	67.4125	183.4182	27.6757	66.0869	6.6811	0.1277
MCEKF	30.0233	67.4515	183.7159	27.6750	66.0893	6.6787	0.3502
MCUKF	30.0049	67.4062	183.3980	27.6679	66.0696	6.6521	0.3889

## Abbreviations

The following abbreviations are used in this manuscript:

SINS	Strap-Down Inertial Navigation System
CNS	Celestial Navigation System
MCC	Maximum Correntropy Criterion
MCUKF	Maximum Correntropy Unscented Kalman Filter
MCEKF	Maximum Correntropy Extended Kalman Filter
UKF	Unscented Kalman Filter
HKF	Huber Kalman Filter
IEKF	Iterated Extended Kalman Filter
NIF	Nonlinear Iterated Filter
PLF	Posterior Linearization Filter
